# Factors Associated with Poor Self-Rated Health among Older Women Living Alone

**DOI:** 10.3390/ijerph191811182

**Published:** 2022-09-06

**Authors:** Eunha Kim, Hye Young Choi

**Affiliations:** 1 College of Nursing, Catholic University of Pusan, 57, Oryundae-to Geumjung-gu, Busan 46252, Gyeongsang-do, Korea; 2 Department of Nursing, Kangwon National University, 346, Hwangjo-gil, Dogye-eup, Samcheok-si 25949, Gangwon-do, Korea

**Keywords:** aged, living alone, self-rated health

## Abstract

Background: The study aimed to identify the differences in self-rated health (SRH) and the factors associated with poor SRH among older women who live alone, based on their sociodemographic and health characteristics. Methods: The sample consisted of 812 older women living alone and was obtained from the Seventh Korean National Health and Nutrition Examination Survey (Ⅶ-1, Ⅶ-2, Ⅶ-3). Complex sample analysis was performed using the independent *t*-test, the Chi-square test, and multiple logistic regression in SPSS/WIN 24.0 program. Results: The study found that SRH status in older women living alone differed according to age, education level, income, occupation, walking practices, sleeping hours, perceived stress, experiencing depression, diabetes, arthritis, and restrictions in daily functions. As the factors associated with poor SRH among older women living alone, a lower education level (OR = 1.89, CI = 1.19–3.02), higher perceived stress (OR = 4.92, CI = 1.84–13.16), experiencing arthritis (OR = 1.52, CI = 1.07–2.16), and higher restrictions in functioning (OR = 6.20, CI = 4.01–9.59) increased the likelihood of rating the poor SRH. Conclusion: SRH is an indicator of overall health status, and physical, psychological, and economic factors affect each other. Therefore, it is necessary to develop effective health education and intervention programs for vulnerable groups, including older women living alone with poor SRH.

## 1. Introduction

### 1.1. Background

The Korean older population aged 65 or over in 2020 is 15.7% of the total population, which is expected to continue to increase in the future, reaching 20.3% in 2025 [1]. During the past 30 years, it has seen a considerable change in household types, along with a rapid increase in the older population; the percentage of the older population that lives alone amounted to 35.1% in 2020 [1]. The older people living alone by age group in their 70s accounted for 44.1%, and by gender, women accounted for 79.1% [2]. 

According to the Korea Institute of Health and Social Affairs, older people living alone showed a lower health status than older people living with family [3]. They had an average of 3.16 chronic diseases, and their self-rated health (SRH) was lower (2.80 out of 5.00) than older people living with family [4]. SHR of older adults is associated with multiple dimensions of current and future health, including morbidity [5], functional health [6], and mortality [7], as well as health care utilization and consumption of medication [8]. In particular, large population studies have shown that older people with poor SRH are associated with socioeconomic status, chronic disease, and health behavior [9]. In older people, having activity of daily living (ADL) limitations or long-term illness is related to poor SRH [10], and many disorders and disabilities are associated with poor SRH in older women living alone [11]. In this way, it is thought to worsen the vulnerable socioeconomic status and unhealthy health behavior SRH of older people.

The Korean older adult living alone was found to have a higher age (70+), lower education level, and lower income compared to the older adult not living alone [4]. The smoking rate of the older people living alone was 92.1%, and the drinking rate was 81.5%, which was higher than that of those non-living alone [3]. This is even more pronounced in the older women living alone, most of whom were below elementary school graduates and lower income levels, such as basic living security, suffering from many chronic diseases, and more physical disabilities [9]. Additionally, they have difficulties in visiting medical institutions due to deteriorating physical functioning and mobility issues [12]. As such, it is thought that the higher vulnerability of socioeconomic and health-related characteristics, the more negatively SRH of the older women living alone. Although many older women who live alone do so in the general community, their socioeconomic characteristics and health-related lifestyles remain poorly understood [13]. 

Looking at the health of older women living alone is a policy concern to achieve health equity, a major social issue in Korea suffering from rapid aging, and to come up with strategies to reduce the gap in health and improve the health of older women living alone. In addition, exploring the factors related to the SRH of older women living alone allows the acquisition of specific data related to their vulnerable socioeconomic status and health problems of them [12]. Therefore, this study aims to analyze poor SRH influencing factors using sociodemographic characteristics and health-related variables as determinants of health in older women aged 65 years or older who live alone in South Korea. We analyzed data from the 7th National Health and Nutritional Examination Survey, which was chosen for its reliability and representativeness of the target population.

In this way, we seek to explore cost-effective strategies to achieve effective management focusing on the target population with poor SRH in older women living alone and the efficient management of expenditure on healthcare and medical expenses and provide basic reference data for the development of policies contributing to interventions that will promote and increase healthy living.

### 1.2. Study Objective

This study aims twofold: (1) to identify the differences in SRH among older women who live alone, based on their sociodemographic and health characteristics; (2) to identify the factors associated with their poor SRH based on these characteristics.

## 2. Materials and Methods

### 2.1. Study Design

This descriptive survey study aims to investigate the Poor SRH-related factors of older women aged 65 years and over who live alone, based on their sociodemographic and health characteristics. It is based on an analysis of secondary data collected in the Korea National Health and Nutrition Examination Survey (KNHANES). 

### 2.2. Study Participants

This study utilized the raw data collected over three years between 2016 and 2018 during the 7th KNHANES that was conducted by the Korea Disease Control and Prevention Agency. The total number of samples collected in the 7th survey was 24,269.

In the first stage of this study, 4956 older people aged 65 years and over were classified as target participants; in the second stage, 2821 older women were selected; in the third stage, 863 older women living alone and without family members were selected; and in the last stage, 812 older women, excluding 51 with missing SRH data, were selected as final participants for the analysis of sociodemographic and health characteristics. 

### 2.3. Variables

The independent variables included sociodemographic and health characteristics, while the dependent variable was SRH. The specific details are as follows. 

#### 2.3.1. Self-Rated Health (SRH)

SRH was measured on a scale of five levels based on the participant’s response to the question of how they would typically rate their health: “Very good”, “Good”, “Moderate”, “Poor”, and “Very poor”. The first three responses, “Very good”, “Good”, and “Moderate”, were grouped as “Moderate or higher”, while the last two, “Poor” and “Very poor”, were grouped as “Poor” according to the study objective.

#### 2.3.2. Sociodemographic Characteristics

The study participants were divided into two age groups: “from 65 to 74 years old” and “75 years old and older”. Educational level was divided into “Elementary school graduate and below” and “Middle school graduate and above”. Income level was divided into “lower” and “lower-middle or higher” based on the household income quartile (low, mid-low, mid-high, or high), and occupational status was divided into either “yes (Employed)” or “no (Unemployed)”.

#### 2.3.3. Health Characteristics

Regarding alcohol consumption, lifelong abstainers or those who had not drunk alcohol for the last 12 months were classified as “non-drinkers”, while the rest of the participants who had consumed alcohol in the past 12 months were classified as “drinkers”. The current smoking habits of the participants were divided into two categories: “smokers” and “non-smokers”. Regarding the practice of regular walking, the participants who answered “at least 5 days a week” and “at least 30 min a day” to the questions, “On how many days have you walked for at least 10 min in the last week?” and “Of these days, how long do you usually walk in a single day?” were classified as those who practiced regular walking, while the rest of the participants were classified as those who did not practice regular walking. The frequency of the participants’ breakfast consumption was divided into two categories: “5–7 times” and “less than 4 times” per week for the last 12 months. The participants were questioned about the average daily hours of sleep they were able to get during the week, and the responses were divided into two categories: the recommended sleep duration of “5–9 h” and “less than 5 h or more than 9 h”. 

The participants’ responses concerning perceived stress levels were divided into two categories, “a lot” and “a little”, depending on the level of stress they perceived to be under in their daily lives. The participants were questioned as to whether they had been diagnosed with diseases such as depression, hypertension, diabetes, and arthritis, and their responses were divided into two categories, “yes” and “no”, depending on whether the applicable disease was present among those who have been diagnosed with the respective disease. Restrictions in daily functions were also assessed, and the participants’ responses were divided into two categories. “yes” and “no”, depending on whether their activities of daily living (ADL) or social activities were restricted due to their health issues or physical or psychological disabilities. 

### 2.4. Data Collection

The two-stage stratified cluster sampling methods with enumeration district and household as primary and secondary sampling were applied to the KNHANES to ensure the representativeness of the sample. The survey was conducted in the mobile health examination stations by the field staff who conducted one-on-one interviews or by self-completion of questionnaires by the participants. In the case of vulnerable persons with poor health status, such as severe cognitive impairment, the proxy persons participated in the interview together and answered the questionnaires. The vulnerable individuals without proxy persons were inevitably excluded from the study sample. The survey field staff included professionals with relevant backgrounds, such as nurses, health science majors, and nutritionists, who surveyed completion of the appropriate training and on-site quality control [14]. 

### 2.5. Ethical Considerations

Anonymized raw data were collected to ensure the confidentiality of the households and individuals that were surveyed following the predetermined procedures such as consenting to the use of personal information through the KNHANES website (https://knhanes.cdc.go.kr). The study was conducted according to the guidelines of the Declaration of Helsinki, and to perform the secondary data analysis, the exemption approval from the institutional review board of the Kangwon National University was obtained (approval number KWNUIRB-2022-02-011). 

### 2.6. Data Analysis

Analysis of data from a complex sample design was performed using the SPSS/Win 24.0 software (SPSS Inc., Chicago, IL, USA) according to the “Guidelines for Analysis of Raw Data from the Korea National Health and Nutrition Examination Survey” [14]. The complex sampling method was used by reflecting strata, clusters, and weight in consideration of the sample design characteristics. The strata are the integration of the design stratum for variance estimation, etc., and the cluster was defined as the enumeration district, the primary extraction unit in the sample design. For weight, the combined weight by selecting a weight corresponding to the type of health survey and multiplying it by the ratio of each survey’s enumeration district number in 2016, 2017, and 2018 year was finally used.

The weighted percentages, means, and standard errors were calculated for the items in participants’ sociodemographic and health characteristics, while the complex sample *t*-test and complex sample *χ*^2^-test were performed to analyze the differences in SRH based on the participants’ sociodemographic and health characteristics. Finally, analysis based on complex samples and multiple logistic regression was performed to identify poor SRH factors based on the participants’ sociodemographic and health characteristics. All statistical significance levels were set to *p* < 0.05. 

## 3. Results

### 3.1. Differences in SRH Based on the Sociodemographic Characteristics of Older Women Living Alone

The SRH of older women living alone was reported as “moderate or higher” in 55.8% of the participants, and as “poor” in 44.2% of them, with statistically significant differences based on age (*χ*^2^ = 607.88, *p* < 0.001), an educational level (*χ*^2^ = 29.85, *p* < 0.001), income level (*χ*^2^= 10.57, *p* = 003), and employment status (*χ*^2^ = 23.07, *p* = 030) (Table 1). Specifically, it found that for those in the age group of 65–74 years old, with an educational level of middle school graduate or higher, with an income level of lower-middle or higher, and who were employed, the proportion of participants who reported their SRH as “moderate or higher” was higher than that of those who rated their health status as “poor” (Table 1).

### 3.2. Differences in SRH Based on the Health Characteristics of Older Women Living Alone

There were statistically significant differences in the SRH of older women who lived alone, depending on whether they practiced regular walking (*χ*^2^ = 22.81, *p* < 0.001), their sleep duration (*χ*^2^ = 5.21, *p* = 0.034), their perceived level of stress (*χ*^2^ = 18.79, *p* < 0.001), the presence of depression (*χ*^2^ = 4.76, *p* = 0.040), the presence of diabetes (*χ*^2^ = 10.74, *p* = 0.003), the presence of arthritis (*χ*^2^ = 15.24, *p* < 0.001), and restrictions in daily functions (*χ*^2^ = 131.34, *p* <.001) (Table 2). We found that for older women practicing the physical activity of regular walking, sleeping from 5 to 9 h, with low perceived stress and without depression, diabetes, arthritis, or restriction in daily functions, the proportion of participants who reported their SRH as “moderate or higher” was higher than that of those who rated their health status as “poor” (Table 2). 

### 3.3. Poor SRH Factors of Older Women Living Alone

To analyze the factors related to the poor SRH of older women who live alone, multiple logistic regression was performed by inputting independent variables that were identified to be statistically significant (*p* < 0.05) in the difference test. Here, 11 variables, including age, education level, income level, employment status, walking practice, sleep duration, perceived stress, depression, diabetes, arthritis, and restrictions in daily functioning, were entered as dummy variables. The sociodemographic and health characteristic variables in the regression model were statistically significant and showed an explanatory power of 30.0% (F = 11.51, *p* < 0.001). Here, education level, perceived stress, the presence of arthritis, and restrictions in daily functions were analyzed as significant poor SRH factors (Table 3). 

More specifically, older women with an education level of elementary or lower are 1.89 times more likely to have poor SRH than those in middle school or higher (OR = 1.89, CI = 1.19–3.02), while the likelihood of poor SRH was 4.92 times higher in older women with “a lot of” stress than those with “a little” stress (OR = 4.92, CI = 1.84–13.16). The likelihood of poor SRH was 1.52 times higher in older women with arthritis than those without arthritis (OR = 1.52, CI = 1.07–2.16), while it was also 6.20 times higher in older women with restrictions in daily functions than those without restrictions in daily functions (OR = 6.20, CI = 4.01–9.59) (Table 3).

## 4. Discussion

This study performed a secondary analysis based on the data collected in the KNHANES (7th) to examine the differences in SRH based on the sociodemographic and health characteristics of older women living alone, and the SRH factors were analyzed through a logistic regression model. Through the analysis, we found that SRH status differed according to the sociodemographic variables (age, education level, income level, and employment status) and health characteristics of older women living alone (regular walking, sleep duration, perceived level of stress, presence of depression, presence of diabetes, presence of arthritis, and restrictions in daily function). As for the factors influencing the poor SRH, restrictions in daily functions, perceived level of stress, education level, and presence of arthritis were statistically significant, and the explanatory power of the variables was 30.0% (*p* < 0.001). Among the analysis results of this study, we focused on the factors influencing SRH, and variables showing significant differences in sociodemographic and health characteristics were also included in the discussion. Because it was variable to be considered to reveal their multilateral vulnerability and enhance the intervention plan for older women living alone.

In total, 44.2% of subjects in this study showed poor SRH, which was higher than 19.9% of older Korean people [1]. In this study, 78.3% of subjects showed an education level below elementary school graduation, 81.3% had a lower income level, and 73.2% had unemployed. This showed a difference from the results of our study, according to the survey of older people in Korea [4], in which 31.7% were elementary school graduates and 49.5% were unemployed. In addition, 30.7% of the subjects practiced walking exercises, 6.8% experienced depression, 61.7% suffered from hypertension, and 25.9% suffered from diabetes. However, a survey of older Korean people in 2020 [1] showed that 39.9% practiced walking exercises, 5.4% were depressed, 57.9% had high blood pressure, and 21.6% had diabetes. Through this, the older women living alone had a lower practice of walking exercise, a higher level of depression, and a higher rate of suffering from hypertension and diabetes than the entire older population in Korea. Therefore, older women living alone in Korea are more socially and economically vulnerable due to poverty, lack of social support, personal restrictions, and physical damage, suggesting that more attention and intervention are needed [9].

Poor SRH was also 6.20 times higher in older women with restrictions in daily functions than those without restrictions in daily functions. A study reported that more restrictions on daily activities were associated with poor SRH in older people in Korea, which is partially supported by the results of this study [15]. In a study that analyzed the functional impairment of older people living alone based on the 2017 National Survey of Older Koreans [4], it was reported that restrictions in daily functioning that interfered with the independent performance of the ADL negatively affected the SRH of older women living alone. These results suggest that musculoskeletal disease, including joint problems, are a significant risk factor for older women’s restrictions in daily functions and that the education level of the older women who live alone is a factor affecting not only restrictions in daily functions but also their overall health status [16]. SRH is an indicator of overall health, and since physical, psychological, and economic factors influence one another, an integrated management approach is required. The findings emphasize the necessity for the development of effective health education and intervention programs for vulnerable socioeconomic groups, including older women who live alone. 

Poor SRH was 4.92 times higher in older women with “a lot of” stress than those with “a little” stress. This is consistent with the results of previous studies [17], which reported that higher perceived stress was associated with poorer SRH. Older women living alone experience more psychological, physical, and social stress due to their lower levels of education and lower income levels compared with their male counterparts. They are particularly vulnerable to stress due to a lack of social support, such as family members or social capital [16]. The older people who live alone in rural areas, with inferior access to social welfare services, were found to experience higher levels of stress due to a lack of leisure activities [18]. Consequently, these results indicate that psychosocial resources through interactions with the community surrounding the older people who live alone play a significant role in relieving mental stress. It is necessary to evaluate the social capital available to the older women who live alone and initiate and enhance a sense of belonging in the local community for those who have limited access to social networks and welfare services to develop and strengthen psychological and social capital and relieve their stress.

Older women living alone with an education level of elementary or lower are 1.89 times more likely to have poor SRH than those in middle school or higher, which is partially consistent with the results of previous studies [17,19] reporting a significant difference in SRH based on the education level of older women. Choi and Lee [19] reported that the level of education strongly affected the health of older people: the lower the education level, such as elementary school graduation or below, the higher the inequality in SRH. The higher the education level, the more social resources are accessible and utilized, such as health information and medical service use networks; thus, a more positive self-assessment of older people’s health was reported in participants with a high level of education [15]. Conversely, poor SRH was reported among participants with a low level of education who lack support systems, such as family members, as they experience difficulty in acquiring and internalizing health information. Additionally, they also experience problems in practicing health promotion behaviors, such as stopping unhealthy habits [20]. Based on these results, we conclude that education level affects the acquisition of social capital by older women who live alone. A lower level of education was found to hurt the process of acquiring social capital and resources related to health. Therefore, to alleviate the inequality experienced by these older women living alone, a strategy is needed to form a network of support in the local community by which they can strengthen their ability to utilize services presented by the public sector. 

Poor SRH was 1.52 times higher in older women with arthritis than those without arthritis; the results of this study are supported by a previous study [17] that utilized the data from the KNHANES (from 2016 to 2018) and reported that older women with one or more chronic diseases perceived their SRH as poor. Approximately 94.2% of the older people living alone suffered from chronic diseases in South Korea [1]. Arthritis was found to be the second most common disease (44.5%) after high blood pressure (60.2%) among older women living alone [21]. Older women often experience pain as a symptom of arthritis, and treatment for this condition is difficult. In chronic diseases such as arthritis, it is important to prevent a worsening of symptoms and to try and minimize complications through self-management and self-monitoring, especially about changes in their symptoms, rather than aiming for a cure [12]. Therefore, it is necessary to develop and provide programs for the education of individuals living with such illnesses for them to acquire knowledge on arthritis management and emergency response methods and to apply this knowledge to their real-life circumstances. Given that non-face-to-face education is the default manner of training following the restrictions imposed under the coronavirus disease 2019 pandemic, the specific circumstances of the older people, in particular their limited access to information and restricted mobility, should be considered in the development of these programs, while encouraging practical disease management by increasing the types of user-oriented services such as home-visits. 

In this study, there were significant differences in the poor SRH of the older women living alone depending on the low income, unemployment, and not walking exercise. This is supported by the finding [17] that economically active older people rate their SRH to be good, as well as another previous study that quantified the effect size of high income from a meta-analysis of the SRH of older people [22]. These results indicate that the SRH of older women who live alone is not independent of the economic context. Recently, the need to secure access to social capital has been emphasized to improve the health and quality of life of older women who live alone. This is a consequence of their role in society has diminished due to low income and decreased interpersonal relationships [20]. Social capital is a social relationship established through interactions between people: a network of neighbors, relatives, religious groups, etc., can serve as a contributor in terms of preventing SRH in older women who live alone from rapid deterioration. However, in South Korea, the social network of older women living alone is rarely extensive. Therefore, it is necessary to establish effective alternatives through studies that examine the individual status of acquiring social capital for older women living alone by comparing the characteristics of the older people who possess abundant social resources and those who do not and then analyzing the factors that affect the acquisition of social capital. 

SRH of older women living alone who walked from six to seven days a week was significantly higher than that of those who did not walk at all [17], which is partially consistent with the findings of this study. Regular walking helps lower one’s resting heart rate and blood sugar level, relieves subjective stress and depressive symptoms, and can help to prevent chronic conditions such as cardiovascular disease, diabetes, and obesity [23]. It also effectively reduces body mass index and decreases annual medical expenses [24]. Therefore, it is necessary to develop a walking program that is tailored to suit the needs and characteristics of the vulnerable older group, especially as the frequency of walking for exercise purposes was low among older people with low levels of education. In addition, the need for health education to motivate residents to practice walking on their own and induce changes in residents’ awareness of the issue of walking has been emphasized [15]. Considering that older women living alone tend to have lower levels of education than older men and that older women are usually financially disadvantaged, walking for exercise is a suitable and convenient health-promoting activity for older women because it is inexpensive and requires less time and physical strength than other forms of exercise. Thus, it is necessary to motivate older women living alone to participate in walking and other forms of exercise to prevent chronic diseases and promote healthy habits. 

SRH of older women living alone with less 5 h or more 9 h of sleep, depression, and diabetes was poorer. A previous large population study [25] analyzed the effect of duration of sleep on SRH among older people, which is similar to this study. Our result was similar to a previous study that showed that poor SRH is associated with a higher prevalence of depression in older adults [26]. Other studies reported that older adults with diabetes have a higher prevalence of depressive symptoms compared with those without the condition [27]. Therefore, it is necessary to prevent diabetes-associated multimorbidity and depressive symptoms to improve SRH. 

This study analyzes individual health determinants for older women who live alone based on representative data collected in the KNHANES. The results of this study are significant in that they confirm the need for older women who live alone to acquire social capital in the local community to improve their SRH, especially as these women are vulnerable in terms of education level and economic status. However, the present study is not without its limitations. First, it is difficult to understand the causal relationships between the SRH factors for older women who live alone as the research is designed as a cross-sectional study. Second, SRH was assessed through a survey using a questionnaire and not based on a physician’s judgment. Thus, it is possible that the appropriate assessment was not conducted for those individuals with severe cognitive impairment. We also need to consider the possibility of having excluded individuals with severely deteriorated health conditions or cognitive decline from participation in the survey.

## 5. Conclusions and Recommendations

The SRH of the older women living alone in Korea was related to various factors. Lower education levels, higher stress perception, chronic diseases such as arthritis, and restrictions on daily activity increased the vulnerability of older women living alone. In particular, since the low socioeconomic level is likely to adversely affect the SRH of older women living alone, policy considerations reflecting this are required. Therefore, it is very important to provide early screening (i.e., active screening of arthritis, stress, and restriction of daily activity) and timely intervention (i.e., consultation with rehabilitation services and mental health professionals) to improve the SRH in vulnerable older women living alone. Based on the above results, we present the following recommendations. First, a health care program needs to be developed and conducted, taking into account the educational level of the older women living alone. Second, since the SRH of older women living alone is affected by physical conditions and restrictions in daily functions, to achieve an improvement in their health, it is necessary to develop services that include various contents such as health care, exercise, counseling, education, leisure time utilization, and participation in social activities. Third, the SRH of older women living alone is greatly affected by psychological, mental, and social factors; thus, a follow-up study should be conducted to consider these factors. Fourth, since the present study is a cross-sectional study with limitations in identifying causal relationships, we suggest that a longitudinal study to examine SRH factors be carried out.

## Figures and Tables

**Table 1:** Differences in self-rated health by sociodemographic characteristics.

Variables			Total	Self-Rated Health		*χ*^2^ or t	*p*
				Moderate or Higher (*n* = 453)	Poor (*n* = 359)		
			*n*^†^ (% ^‡^) or M ± SE ^§^	*n*^†^ (% ^‡^) or M ± SE^§^	*n*^†^ (% ^‡^) or M ± SE ^§^		
Socio demographics	Age	65–74	352 (38.9)	221 (45.1)	131 (31.0)	607.88	<0.001
		≥75	460 (61.1)	232 (54.9)	228 (69.0)		
		Mean ± SD	75.19 ± 0.18	74.60 ± 0.24	75.79 ± 0.289	262.56	0.003
	Educational level *	≤Elementary	635 (78.3)	328 (71.3)	307 (87.4)	29.85	<0.001
		≥Middle school	163 (21.7)	120 (28.7)	43 (12.6)		
	Income level *	Lower	648 (81.3)	336 (75.5)	312 (88.8)	23.07	<0.001
		≥Mid-lower	159 (18.7)	116 (24.5)	43 (11.2)		
	Occupational status *	Yes	206 (26.8)	13.7 (31.2)	69 (20.9)	10.57	0.003
		No	592 (73.2)	311 (68.8)	281 (79.1)		

M = mean; SE = standard error; * missing values were excluded; ^†^ non-weighted sample size; ^‡^ weighted %; ^§^ weighted mean ± standard error.

**Table 2:** Differences in self-rated health by health characteristics.

Variables			Total	Self-Rated Health		*χ* ^2^	*p*
				Moderate or Higher (*n* = 453)	Poor (*n* = 359)		
			*n*^†^ (% ^‡^) or M ± SE ^§^	*n*^†^ (% ^‡^) or M ± SE ^§^	*n*^†^ (% ^‡^) or M ± SE ^§^		
Health status	Drinking *	Yes	288 (36.2)	175 (39.4)	113 (32.0)	4.72	0.053
		No	515 (63.8)	276 (60.6)	239 (68.0)		
	Current smoking *	Yes	21 (2.6)	10 (2.2)	11 (3.0)	0.53	0.496
		No	781 (97.4)	440 (97.8)	341 (97.0)		
	Walking * (for the last week)	Yes	236 (30.7)	158 (37.6)	78 (21.9)	22.81	<0.001
		No	559 (69.3)	286 (62.4)	273 (78.1)		
	Frequency of breakfast * (for last week)	≤4	74 (9.7)	37 (8.5)	37 (11.3)	1.67	0.221
		5~7	686 (90.3)	391 (91.5)	295 (88.7)		
	Sleeping hours * (per day)	5~9	671 (84.4)	392 (87.0)	279 (81.1)	5.21	0.034
		<5 or >9	121 (15.6)	55 (13.0)	66 (18.9)		
	Perceived stress *	A lot	37 (4.0)	9 (1.4)	28 (7.4)	18.79	<0.001
		A little	763 (96.0)	441 (98.6)	322 (92.6)		
	Depression *	Yes	54 (6.8)	25 (5.1)	29 (9.0)	4.76	0.040
		No	756 (93.2)	427 (94.9)	329 (91.0)		
	Hypertension	Yes	499 (61.7)	269 (60.1)	230 (63.7)	1.13	0.383
		No	313 (38.3)	184 (39.9)	129 (36.3)		
	Diabetes mellitus	Yes	212 (25.9)	96 (21.5)	116 (31.6)	10.74	0.003
		No	600 (74.1)	357 (78.5)	243 (68.4)		
	Arthritis	Yes	364 (44.1)	172 (38.1)	192 (51.8)	15.24	<0.001
		No	448 (55.9)	281 (61.9)	167 (48.2)		
	Restrictions in daily functions *	Yes	54 (6.8)	25 (5.1)	29 (9.0)	131.34	<0.001
		No	756 (93.2)	427 (94.9)	329 (91.0)		

M = mean; SE = standard error; * missing values were excluded; ^†^ non-weighted sample size; ^‡^ weighted %; ^§^ weighted mean ± standard error.

**Table 3:** Associated factors of poor self-rated health.

	Variables		Self-Rated Health (*n* = 812)		
			OR	95% CI	*p*
Socio demographics	Age	65∼74	0.72	0.50–1.03	0.075
		≥75	1		
	Educational level	≤Elementary	1.89	1.19–3.02	0.008
		≥Middle school	1		
	Income level	Low	1.59	0.99–2.57	0.057
		≥Mid-lower	1		
	Occupational status	Yes	0.70	0.46–1.05	0.085
		No	1		
Health status	Walking (for the last week)	Yes	0.70	0.48–1.03	0.072
		No	1		
	Sleeping hours (per day)	5~9	0.68	0.42–1.11	0.121
		<5 or >9	1		
	Perceived stress	Much	4.92	1.84–13.16	0.002
		A little	1		
	Depression	Yes	0.85	0.39–1.86	0.676
		No	1		
	Diabetes mellitus	Yes	1.45	0.97–2.17	0.074
		No	1		
	Arthritis	Yes	1.52	1.07–2.16	0.021
		No	1		
	Restrictions in daily functions	Yes	6.20	4.01–9.59	<0.001
		No	1		
	F (*p*)		11.51 (<0.001)		
	R^2^		0.300		

## Data Availability

3rd Party Data Restrictions apply to the availability of these data. Data was obtained from [Korea Centers for Disease Control and Prevention] and are available [from Department of Health and Nutrition Examination Survey Analysis /at https://knhanes.kdca.go.kr/knhanes/sub03/sub03_02_05.do] (accessed on 26 February 2021) with the permission of [Korea Centers for Disease Control and Prevention].
